# Tipping point analysis in network meta-analysis

**DOI:** 10.1017/rsm.2025.24

**Published:** 2025-06-16

**Authors:** Zheng Wang, Thomas A. Murray, Wenshan Han, Lifeng Lin, Lianne K. Siegel, Haitao Chu

**Affiliations:** 1 Department of Biostatistics and Research Decision Sciences, Merck & Co., Inc., Rahway, NJ, USA; 2 Division of Biostatistics and Health Data Science, School of Public Health, University of Minnesota, Minneapolis, MN, USA; 3 Department of Population and Community Health, University of North Texas Health Science Center, Fort Worth, TX, USA; 4 Department of Epidemiology and Biostatistics, University of Arizona, Tucson, AZ, USA; 5 Statistical Research and Data Science Center, Pfizer Inc., New York, NY, USA

**Keywords:** correlation between multiple treatments, network meta-analysis, robustness of research conclusion, sensitivity analysis, tipping point analysis

## Abstract

Network meta-analysis (NMA) enables simultaneous assessment of multiple treatments by combining both direct and indirect evidence. While NMAs are increasingly important in healthcare decision-making, challenges remain due to limited direct comparisons between treatments. This data sparsity complicates the accurate estimation of correlations among treatments in arm-based NMA (AB-NMA). To address these challenges, we introduce a novel sensitivity analysis tool tailored for AB-NMA. This study pioneers a tipping point analysis within a Bayesian framework, specifically targeting correlation parameters to assess their influence on the robustness of conclusions about relative treatment effects. The analysis explores changes in the conclusion based on whether the 95% credible interval includes the null value (referred to as the *interval conclusion*) and the magnitude of point estimates. Applying this approach to multiple NMA datasets, including 112 treatment pairs, we identified tipping points in 13 pairs (11.6%) for *interval conclusion change* and in 29 pairs (25.9%) for *magnitude change* with a threshold at 15%. These findings underscore potential commonality in tipping points and emphasize the importance of our proposed analysis, especially in networks with sparse direct comparisons or wide credible intervals for correlation estimates. A case study provides a visual illustration and interpretation of the tipping point analysis. We recommend integrating this tipping point analysis as a standard practice in AB-NMA.

## Highlights

**What is already known**

- Network meta-analysis (NMA) facilitates simultaneous assessment of multiple treatments by integrating both direct (from studies comparing treatments directly) and indirect evidence (from trials that share a common treatment).
- A major challenge in NMA is the limited availability of direct comparisons between all treatment pairs, often due to the resource constraints and evolving nature of control arms in randomized controlled trials (RCTs).
- This data sparsity complicates the accurate estimation of correlations between the random effects of multiple treatments within each study when using arm-based NMA models.

**What is new**

- To address the challenges brought by sparse data in NMA, we propose a novel tipping point analysis to assess the robustness of treatment effect conclusions, focusing on the changes of statistical conclusion based on the credible interval and effect magnitude, by varying correlation strengths between the random effects of treatments in an arm-based NMA (AB-NMA).
- When applied to 14 NMA datasets, the results reveal potential commonalities in tipping points, highlighting the importance of our proposed analysis, especially in networks with sparse direct comparisons or wide credible intervals for estimated correlations.

**Potential impact for RSM readers**

- The proposed tipping point analysis complements the AB-NMA modeling and provides a novel tool for researchers to assess the reliability of conclusions regarding treatment effect. It ensures that the findings are not only supported by the analysis but also robust to variations in correlation assumptions.

## 1 Introduction

Network meta-analysis (NMA), also known as mixed treatment comparisons meta-analysis or multiple treatments meta-analysis, has emerged as a powerful tool for synthesizing evidence across multiple studies, especially when multiple treatments are being compared and direct comparisons among all treatments are not available.^1^ ^,^ ^2^ Traditional pairwise meta-analyses limit comparisons to two treatments at a time, but NMA extends this framework, allowing for the simultaneous assessment of multiple treatments. By integrating both direct evidence (from studies comparing treatments head-to-head) and indirect evidence (derived from two or multiple studies that have a common treatment or control arm), NMA facilitates a holistic understanding of the treatment landscape. Specifically, direct evidence refers to the evidence that directly compares the two treatments, for example, treatment A vs. B in a randomized controlled trial (RCT), while indirect evidence between A vs. B is provided by comparing A vs. C and C vs. B in two RCTs.^3^ ^–^ ^5^ Borrowing information from indirect evidence under suitable assumptions, such as transitivity and evidence consistency, may yield a more precise evaluation of treatment effects than those synthesized solely from direct evidence in pairwise meta-analysis.^6^

There are two main approaches to conducting an NMA: contrast-based (CB) and arm-based (AB). Each has its own distinct emphasis and underlying assumptions. The CB-NMA operates under the assumption that relative effects, or contrasts, are exchangeable across studies, and focuses its analysis on the estimation of the overall relative effect.^7^ ^–^ ^9^ In contrast, AB-NMA makes an arguably more stringent assumption that the absolute effects are exchangeable across studies, offering the added benefit of estimating both the absolute effects of each treatment and relative effects for each pair of treatments.^10^ ^–^ ^13^ As such, AB-NMA can provide useful information in cost-effectiveness analysis, aiding in the optimization of resource distribution.^14^ Moreover, when compared to CB-NMA, AB-NMA has demonstrated lower sensitivity in the effect estimates to the choice of treatments included in the treatment network.^15^ While the AB-NMA model offers several advantages, it also has certain limitations that warrant consideration. A key limitation is that AB-NMA models can potentially introduce bias due to breaking randomization when there are systematic differences among trials of different designs. This concern has been a subject of debate in the literature.^16^ ^,^ ^17^ However, the practical impact of this limitation may need further investigation to determine whether it poses significant challenges in real-world applications.^18^

A big challenge in NMA is the sparse information from the lack of direct comparisons between all treatment pairs, often attributed to the inherent resource limitations and evolutionary nature of control arms in RCTs. Given financial and operational considerations, an RCT typically compares 2–4 treatments, leading to common scenarios where not all treatments in an NMA are directly compared. Some NMAs even have a “star-shaped” structure, wherein each treatment is directly evaluated relative to a common control and never relative to each other within the same trial.^19^ Such data sparsity makes accurate estimation of the correlation among the study-specific random effects of multiple treatments in AB-NMA challenging.^20^ New methods have been recommended to improve the estimation of the correlation matrix compared to the traditional inverse-Wishart (IW) prior in AB-NMA. These include a separation strategy to separate priors on variances and correlations,^20^ a uniform prior for the correlation parameter with a lower boundary that ensures the resulting correlation matrix is positive definite,^13^ and a variance shrinkage method for variance estimation.^21^ A tipping point analysis may supplement more information about whether the study conclusion is robust across different strengths of the correlation.

A tipping point analysis aims to assess the robustness of research conclusions by investigating how potential alterations to the analysis assumptions or observed data might influence study conclusions substantively.^22^ ^,^ ^23^ Identifying the “tipping point,” i.e., the juncture where a minor change substantively alters a study conclusion, may offer insights into the fragility and reliability of study findings. Tipping point analysis is especially relevant in fields where decisions are informed by data, such as healthcare or economics, as they help researchers and policymakers gauge the confidence they can place in a given result, ensuring that conclusions drawn are not only supported by the analysis but also meaningfully robust to key analysis assumptions.^24^ ^–^ ^29^ Han et al.^30^ recently introduced a tipping point analysis for pairwise meta-analysis, specifically to assess the robustness of conclusion when incorporating single-arm studies. Their analysis, which focused on comparisons involving only two treatments, demonstrated its potential to provide valuable insights that complement conclusions about relative treatment effects. Such tipping point analysis can be further extended to more complex scenarios, such as NMA where multiple treatment comparisons are synthesized jointly.

To the best of our knowledge, no methods have been previously proposed for conducting a tipping point analysis with respect to the assumed correlation structure within an NMA. This article aims to bridge this gap by introducing a novel sensitivity analysis tool tailored for AB-NMA. Within the Bayesian framework, our method searches for tipping points in correlation parameters that alter conclusions about relative treatment effects. These conclusions include: (1) whether the 95% credible interval includes the null value (referred to as the *interval conclusion*) and (2) the magnitude of point estimates. The *interval conclusion* aligns conceptually with the statistical significance conclusion in the Frequentist framework, offering a dichotomized conclusion about the strength of evidence.

We organize the content of this article as follows. The proposed method with detailed Bayesian model specifications and procedure steps is explained in Section 2. Section 3 details some NMA datasets to which we applied the proposed tipping point analysis. Applied examples and their results are presented in Section 4. Finally, a comprehensive discussion is provided in Section 5.

## 2 Statistical analysis methods

### 2.1 AB-NMA model

Before describing the tipping point analysis method, we first present the model specification for the AB-NMA. In an NMA reviewing *N* studies and *K* treatments, let *i* denote the study, 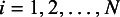 ; let *k* denote the treatment ( 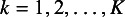 ), and 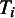 is the subset of treatments investigated in study *i*. In study *i* for treatment *k* ( 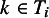 ), let 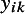 denote the number of events for a binary outcome, 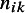 denote the total number of subjects, and 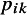 denote the underlying absolute risk. The AB-NMA model with a logit link can be specified as 

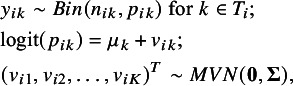

where 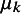 denotes the fixed effect of the treatment *k*, 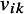 denotes the random effect of the treatment *k* in study *i*, and 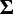 denotes the variance–covariance matrix of the vector of random effects 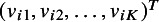 . The population-averaged absolute risk of treatment *k* can be approximated as 

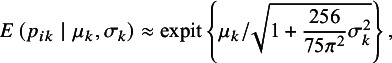

where 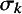 denotes the standard deviation of the random effect of the treatment *k*, and the relative risk (RR), risk difference (RD), and odds ratio (OR) can be calculated based on the absolute risk using consistency equations.^21^ ^,^ ^31^

As recommended by Wang et al.,^21^ we used the separation strategy and the variance shrinkage method to assign weakly informative priors to 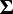 . It can be decomposed as 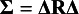 , where 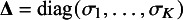 , and 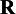 is the correlation matrix. By assuming the exchangeable correlation structure, the correlation matrix is a *K* by *K* matrix as follows: 

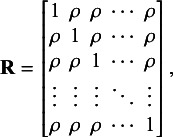

where 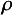 denotes the correlation.

Then, a uniform prior is used for the standard deviation^32^: 

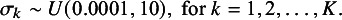

Alternatively, a hierarchical half-Cauchy (HHC) prior for the standard deviation is recommended by Wang et al.^21^: 

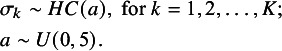

A uniform prior is commonly used for the correlation parameter 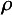 , 

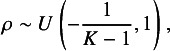

where the lower bound is chosen to guarantee that the correlation matrix 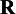 is positive definite. For the fixed effect, 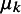 , we assign a non-informative prior: 

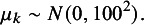



### 2.2 Method for tipping point analysis

Our tipping point analysis focuses on the correlation parameter 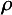 . A tipping point is defined as the value for 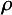 where some substantive conclusion changes, e.g., the *interval conclusion* flips or the change in magnitude of the relative treatment effect exceeds some meaningful threshold. The proposed procedure for searching for a tipping point is as follows:

1. Conduct the NMA with the model specified in Section 2.1 to estimate:
  - Population-averaged point estimate and 95% credible interval of the treatment effect, e.g., RR or RD, for all pairs of treatments;
  - The posterior median, 95% credible interval, and a series of other posterior percentiles for the correlation parameter 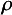 .
  The number of percentiles for 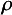 depends on how granular one wants to be when checking for a tipping point. We recommend using the following 11 percentiles of the posterior samples: 1%, 2.5%, 5%, 10%, 25%, 50%, 75%, 90%, 95%, 97.5%, and 99%. These were chosen to include the important posterior percentiles that are commonly reported in the summary of a Bayesian posterior distribution: 2.5%, 25%, 50%, 75%, and 97.5%,^33^ supplemented by additional points to provide more granularity. While a tighter grid of percentiles may provide a more accurate estimate of the tipping point, it is more computationally intensive.
2. Repeatedly conduct the NMA with the model described in Section 2.1, but instead of assigning a prior distribution to 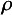 , set 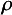 to each value of the estimated percentiles in Step 1. Thus, 11 NMA models with different assumed values for 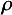 values are fitted separately, and the mean and 95% credible intervals of the treatment effects for all pairs of treatments are obtained in each model. Draw the *interval conclusion* of the treatment effect based on whether the 95% credible interval includes the null value, e.g., the null value is 1 for RR and 0 for RD.
3. Search for a tipping point at the 11 data points based on either of the following criteria:
  - *Interval conclusion* change: An interval conclusion change occurs when the interval conclusion of a treatment effect comparing a pair of treatments based on the result of one or more NMAs in Step 2 is opposite to the interval conclusion for this pair of treatments based on the result of the NMA in Step 1. For example, an interval conclusion can shift from strong evidence supporting the presence of a treatment effect (i.e., 95% credible interval does not include the null value) to weak evidence (i.e., 95% credible interval includes the null value), or vice versa. The 95% credible interval has been widely used for such conclusion because a posterior probability threshold of 0.975 is commonly accepted in determining the strength of evidence. However, alternative thresholds other than 0.975 can be carefully selected depending on the clinical context and specific study objectives.
  - *Magnitude change*: A magnitude change happens when the percent change in the magnitude of the mean treatment effect comparing a pair of treatments in Step 2 relative to the result of the NMA in Step 1 exceeds some meaningful threshold. The choice of thresholds should ideally be informed by clinical expertise and tailored to the specific disease context and primary outcomes. To demonstrate the methodology, in the selected NMA datasets, we considered thresholds of 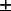 15% and 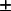 30%, corresponding to low and high thresholds.

**Figure 1:**
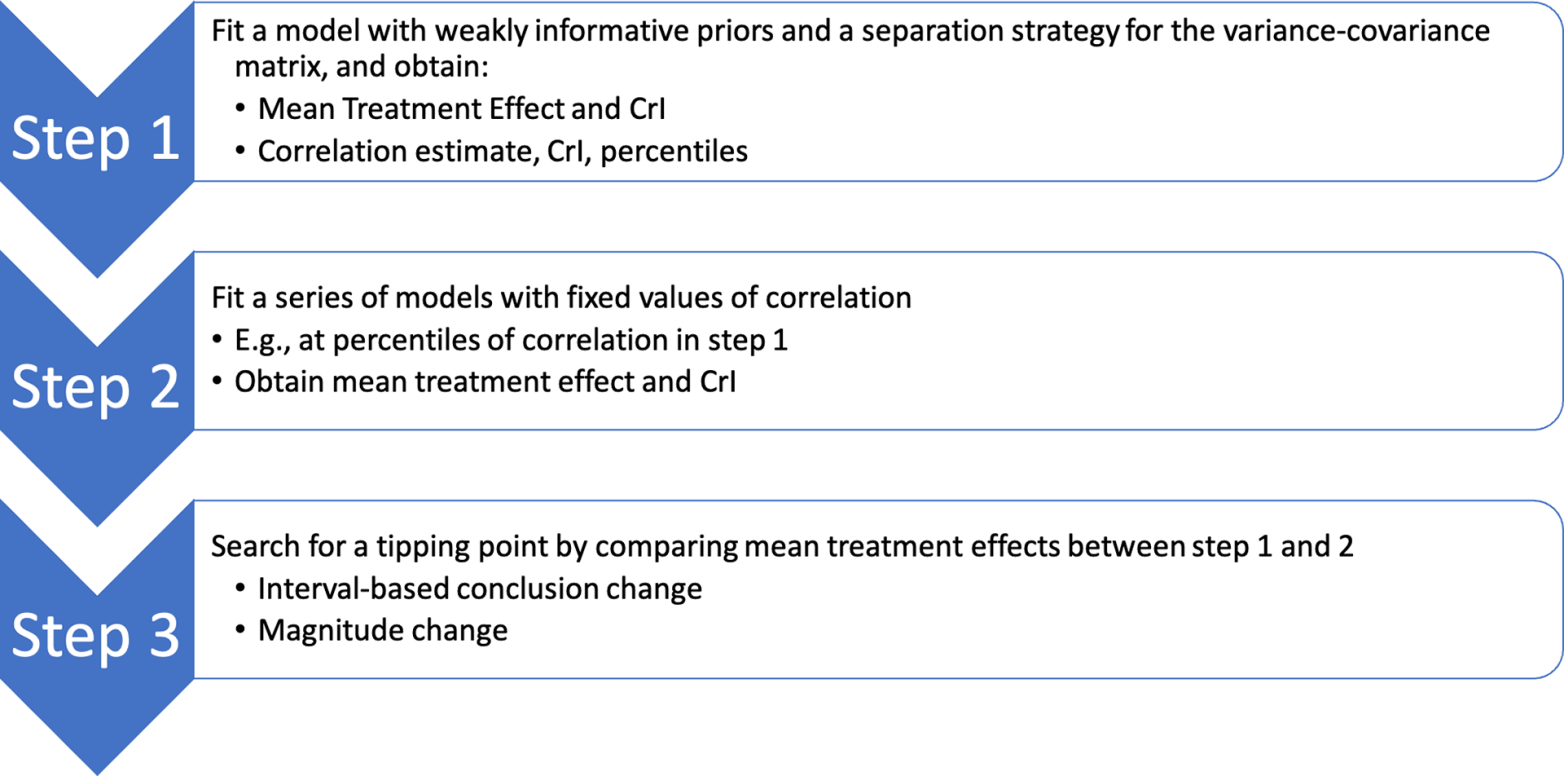
Steps of searching for tipping points in correlation parameters that alter conclusions about relative treatment effects in an NMA.

A schematic summary of the above steps is presented in Figure 1. In Step 2, the models can be executed in parallel for a comprehensive search. Alternatively, to reduce the computational demands, we may also adopt a bisection approach to pinpoint the location of the tipping point by iteratively narrowing a range of search. Starting with a broad range, for example, from 1 to 99 percentiles, we only compare two models fitted with the 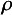 values at the two endpoints of the range. If we observe either an *interval conclusion change* or a *magnitude change*, the search continues and the range is narrowed down in the next iteration; otherwise, the searching process ends, concluding no tipping point.

All analyses were conducted on JAGS version 4.3.0-gcc7.2.0 through the R package “rjags” in R version 4.2.2. The JAGS code for Bayesian models is provided in Appendix 1 of the Supplementary Material.

## 3 Dataset selection

### 3.1 Dataset screening and selection

**Figure 2:**
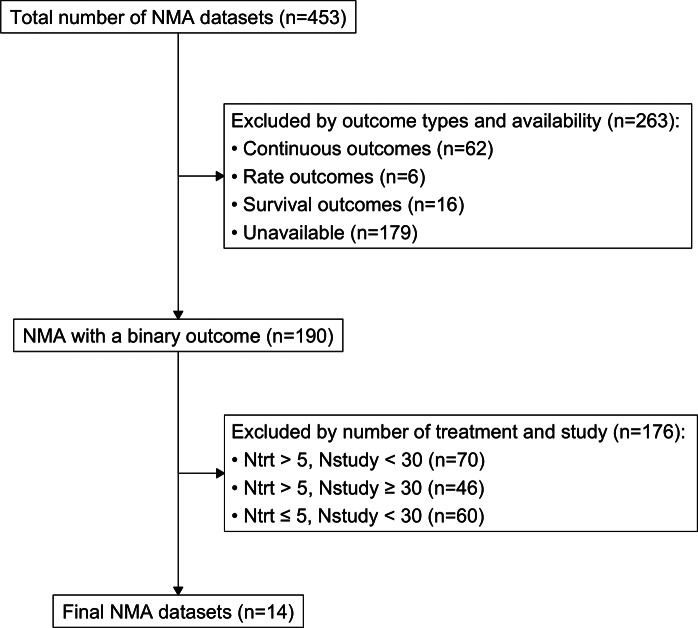
A diagram of the dataset screening process. Note: 453 datasets were extracted from the “nmadb” package in R. All selections were based on the record in the package.

We extracted 453 NMA datasets published between 1999 and 2015^34^ from the R package “nmadb,”^35^ which provides the Application Programming Interface (API) for an NMA database. With computational considerations and with the purpose to include various types of networks with a varied sparsity to demonstrate our method on networks that reflect more typical real-world scenarios, we evaluated NMA datasets with the following inclusion criteria: (1) a binary outcome, (2) 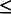 5 treatments, and (3) 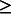 30 studies. The detailed workflow for this screening process is illustrated in Figure 2. Using these criteria, we identified 14 candidate NMA datasets that collectively comprised 790 studies with 355,923 total participants.

The candidate NMA datasets are named after the first author and the year of publication. Table 1 describes each of these datasets in terms of the number of studies, number of treatments, primary outcome, total number of events, total number of subjects, and the median (minimum, maximum) event rate across the studies. These networks encompass a diverse range of therapeutic areas such as cardiovascular (Ribeiro2011), respiratory (Puhan2009, Mills2010, and Eisenberg2008), gastroenterology (Yang2014b and Tadrous2014), neurology and psychiatry (Furukawa2014), hematology and oncology (Wang2015), and infectious disease (Edwards2009b). The median event rates in these studies spanned from 0.00 to 0.88. This broad range indicated the inclusion of both rare and common primary outcomes within the selected networks.

**Table 1:** A summary of selected network meta-analyses

	No. of	No. of	Total events	Median event	
Name of NMA	study	treatment	/ Total subjects	rate (min, max)	Primary outcome
Yang2014b^44^	145	5	10,332 / 12,983	0.82 (0.10, 1.00)	Crohn’s disease recurrence
Mills2010^45^	89	4	10,847 / 29,525	0.36 (0.00, 0.85)	Smoking abstinence
Hutton2012^46^	77	4	368 / 14,165	0.00 (0.00, 0.21)	All-cause mortality
Trikalinos2009^36^	63	4	821 / 26,748	0.01 (0.00, 0.21)	All-cause mortality
Eisenberg2008^47^	61	5	3,908 / 26,730	0.14 (0.01, 0.57)	Continuous smoking abstinence at 12 months
Furukawa2014^48^	48	4	893 / 2,722	0.33 (0.00, 0.90)	The number of patients who responded to depression treatment
Greco2015^49^	46	5	145 / 2,647	0.00 (0.00, 0.32)	Mortality in adult cardiac surgery patients
Tadrous2014^50^	46	5	8,486 / 33,227	0.20 (0.02, 0.64)	Gastrointestinal related adverse events
Moore2005^51^	45	4	5,853 / 11,315	0.45 (0.04, 0.90)	Improved erection dysfunction
Ribeiro2011^52^	39	4	6,462 / 155,185	0.03 (0.00, 0.12)	Cardiovascular prevention
Gafter-Gvili2005^53^	37	5	325 / 2,718	0.08 (0.00, 0.71)	All-cause mortality
Puhan2009^54^	34	5	7,200 / 26,789	0.24 (0.00, 0.62)	Exacerbation in patients with chronic obstructive pulmonary disease
Edwards2009b^55^	30	4	4,263 / 5,133	0.88 (0.51, 1.00)	Effectiveness of beta-lactams for the treatment of hospitalized patients with infection
Wang2015^56^	30	5	1,326 / 6,036	0.20 (0.00, 0.78)	Febrile neutropenia risk for all chemotherapy cycles without adjustment for relative dose intensity

**Figure 3:**
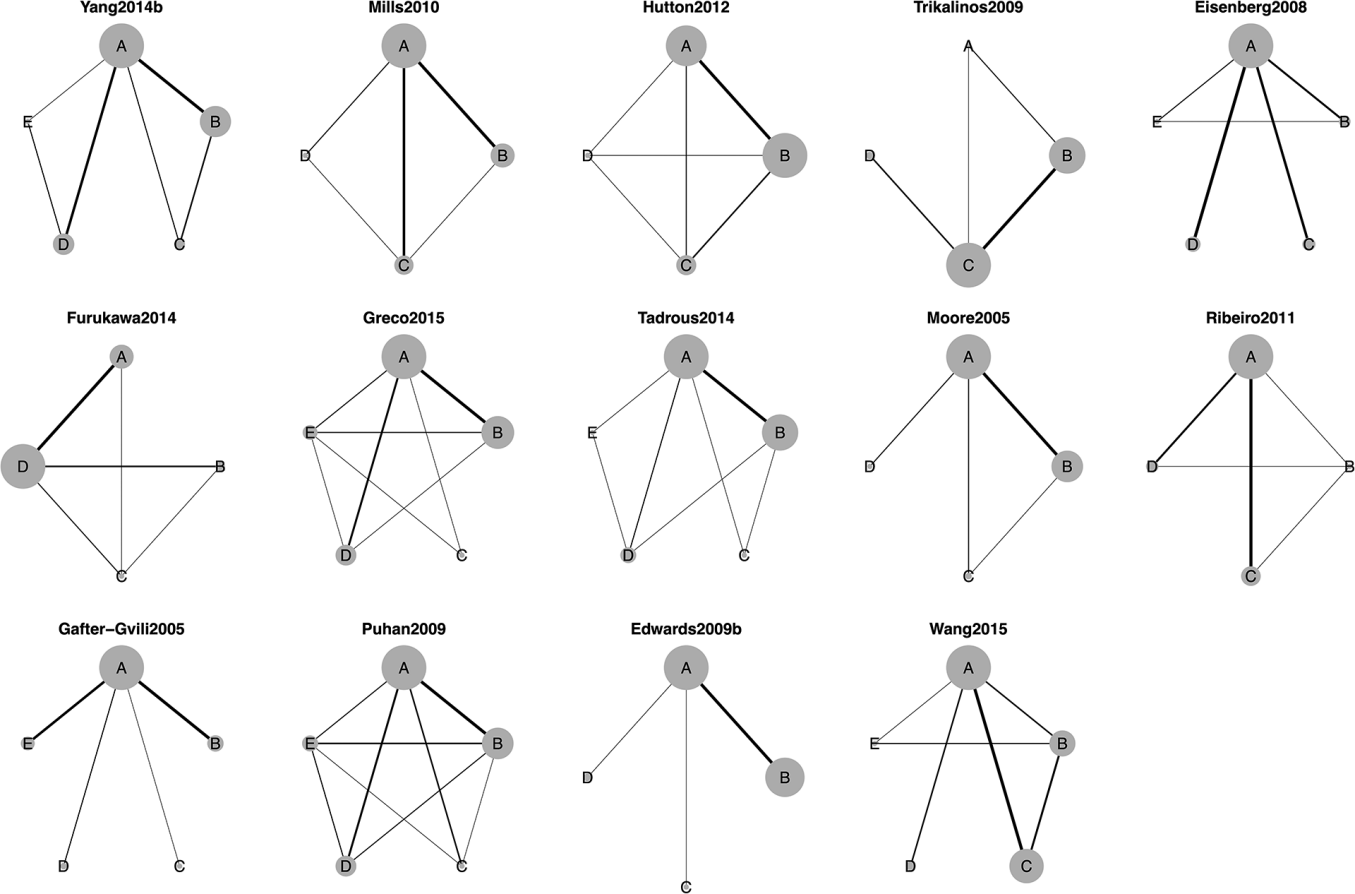
Network plots of selected NMA datasets. Note: The 4th plot in the first row represents the network selected as the case study. The nodes with uppercase letters indicate the distinct treatments in the network, and the edges indicate the direct comparisons in an RCT. The weight of each edge is determined by the number of studies with a direct comparison between the connected treatments.

Figure 3 shows the network plots for the 14 selected NMA datasets. In these plots, the nodes symbolize the treatments and are labeled with uppercase letters, and the edges between nodes indicate direct treatment comparisons. The weight of each edge is determined by the number of studies with a direct comparison between the connected treatments. Additionally, the size of each node is determined by the number of studies containing the specific treatment, offering an intuitive visual representation of the treatment’s prevalence within the network. The selected networks exhibited a diverse range of network plot configurations and sparsity characteristics. For example, Gafter-Gvili2005 and Edwards2009b had high sparsity demonstrated by star-shaped structures, while Hutton2012 and Puhan2009 had low sparsity with well-connected networks. Besides these two types of extreme networks, others showed moderate sparsity with more complex networks with loops and some groups with sparse connections.

**Figure 4:**
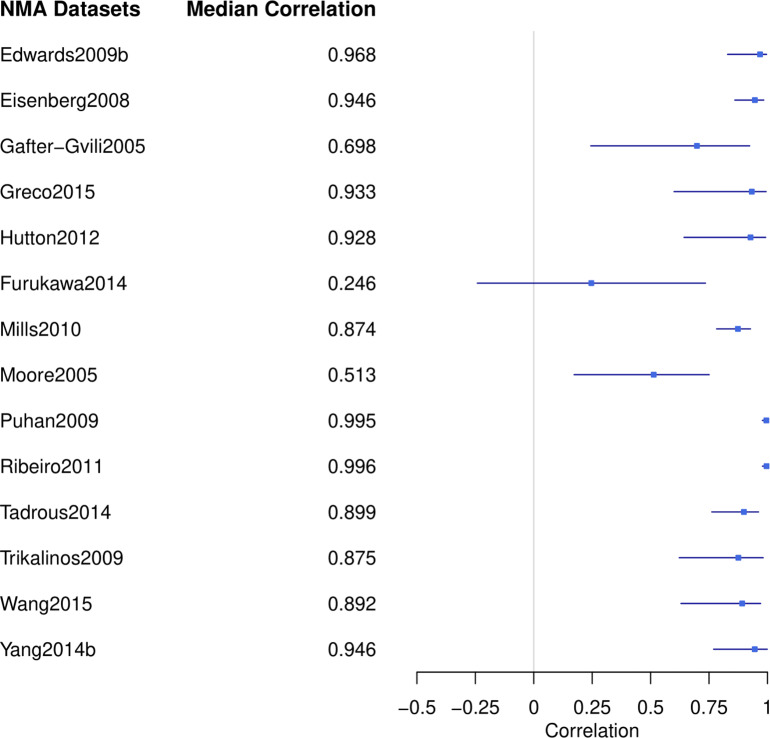
Forest plot of the posterior median correlation estimates based on AB-NMA model in selected NMA datasets.

Figure 4 displays the forest plot of the estimated correlation between treatments across the 14 selected NMA datasets using AB-NMA models as specified in Section 2.1. These networks exhibited a broad range of posterior median correlations, from 0.266 to 0.996. Additionally, the 95% credible intervals of the correlation differed in length, as evidenced by the varying lengths of the blue lines. Such diversity in the datasets reflected the comprehensive range of selected studies and allowed us to demonstrate the applicability of the tipping point analysis across diverse real-world examples.

### 3.2 Dataset of the case study

To illustrate the proposed tipping point analysis in more detail, we selected one network from the selected 14 networks, the Trikalinos2009^36^ NMA dataset, as a detailed case study. The NMA study by Trikalinos et al. from 2009 in the *Lancet* evaluated catheter-based treatments, specifically percutaneous coronary intervention (PCI), for non-acute Coronary Artery Disease (CAD). The study focused on medical therapy (including administration of antiplatelet agents, 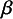 blockers, nitrates, calcium channel blockers, or aggressive lipid-lowering treatment)^37^ and 3 main types of PCI interventions: Percutaneous Transluminal Balloon Coronary Angioplasty (PTCA), Bare Metal Stents (BMS), and Drug-Eluting Stents (DES). There were 4 outcomes of interest: all-cause mortality, myocardial infarction (MI), coronary artery bypass grafting (CABG), target lesion or vessel revascularization (TLR/TVR), and any revascularization. In our case study, we focus on the primary outcome, all-cause mortality.

The Trikalinos2009 NMA included 61 RCTs and a total of 25,388 participants. Since two of the trials, which reported results for 2 separately randomized strata, were entered as 4 distinct entries in the meta-analyses, this NMA dataset comprised 63 unique trial entries and 26,748 subjects. Of the 6 possible pairwise treatment comparisons, this NMA dataset contained trials with direct comparisons for 4 of these; there was no trial with a direct comparison of DES and PTCA, or DES and medical therapy.

In their analysis, Trikalinos et al. used a CB model and assumed the relative effects were exchangeable. In particular, there were inconsistencies in the results based on the direct pairwise comparison and the results based on NMA, which included indirect evidence. The RR of death for PTCA vs. medical therapy evaluated in direct comparisons had an opposite direction compared to the indirect RR of death when evaluated in the NMA. The overall conclusion was that no evidence showed PCI treatments (including PTCA, BMS, and DES) were more effective than medical therapy in treating non-acute CAD.

## 4 Results

### 4.1 Results of the selected NMA datasets

We conducted the proposed tipping point analysis on the 14 NMA studies described in Section 3.1 using RR as the treatment effect measure. The cumulative incidence of a tipping point for *interval conclusion* change and *magnitude change* are shown in Tables 2 and 3, respectively. These counts were arranged in ascending order based on the distance (in percentiles) between the percentile of the fixed 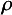 values and the median estimated correlation from the AB-NMA model in Step 1 in Section 2.2.

**Table 2:** A summary of the incidences and the cumulative incidences of *interval conclusion* change tipping point among the 14 selected NMA datasets, with relative risk as the measure of treatment effect

	Count of pairs with	Cumulative incidence of a
Percentile from median (Percentiles)	a tipping point	tipping point (% in 112 pairs)
0% (50%)	2	2 (1.8%)
25% (25%, 75%)	4	6 (5.4%)
40% (10%, 90%)	0	6 (5.4%)
45% (5%, 95%)	3	9 (8.0%)
47.5% (2.5%, 97.5%)	3	12 (10.7%)
49% (1%, 99%)	1	13 (11.6%)

**Table 3:** A summary of the cumulative incidences of the *magnitude change* tipping point among the 14 selected NMA datasets, with relative risk as the measure of treatment effect

Percentile from	Cumulative incidence of a magnitude change tipping point (% in 112 pairs)			
median (Percentiles)	Threshold = 15%	Threshold = 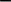 15%	Threshold = 30%	Threshold = 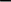 30%
0% (50%)	4 (3.6%)	2 (1.8%)	2 (1.8%)	2 (1.8%)
25% (25%, 75%)	6 (5.4%)	2 (1.8%)	2 (1.8%)	2 (1.8%)
40% (10%, 90%)	20 (17.9%)	10 (8.9%)	4 (3.6%)	2 (1.8%)
45% (5%, 95%)	24 (21.4%)	16 (14.3%)	9 (8%)	6 (5.4%)
47.5% (2.5%, 97.5%)	24 (21.4%)	21 (18.8%)	17 (15.2%)	9 (8%)
49% (1%, 99%)	29 (25.9%)	22 (19.6%)	20 (17.9%)	11 (9.8%)

From the 112 treatment pairs across the 14 NMAs, there were 13 tipping points for *interval conclusion* change. That is, 11.6% of the pairwise treatment comparisons exhibited a tipping point where the *interval conclusion* was altered. When setting the relative difference thresholds for the *magnitude change* tipping point at 15%, 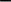 15%, 30%, and 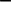 30%, the cumulative incidences were 29, 22, 20, and 11, corresponding to 25.9%, 19.6%, 17.9%, and 9.8%, respectively. Although there were fewer tipping points for the larger magnitude change threshold, there were still several pairwise comparisons across the 14 NMAs that exhibited such magnitude change tipping points. These *interval conclusion* change and *magnitude change* tipping points indicated that the statistical conclusion of relative effect for these treatment pairs may be sensitive to some assumed correlation values within the realm of plausibility.

It is worth noting that among the 14 selected datasets, 8 out of 13 pairs that exhibited an *interval conclusion change* tipping point had limited or no ( 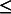 1) direct comparisons, as shown in Figure S1 in Appendix 2 of the Supplementary Material. This underscored the importance of the tipping point analysis when drawing conclusions from NMA for treatment pairs with few direct comparisons.

### 4.2 Results of the case study

**Figure 5:**
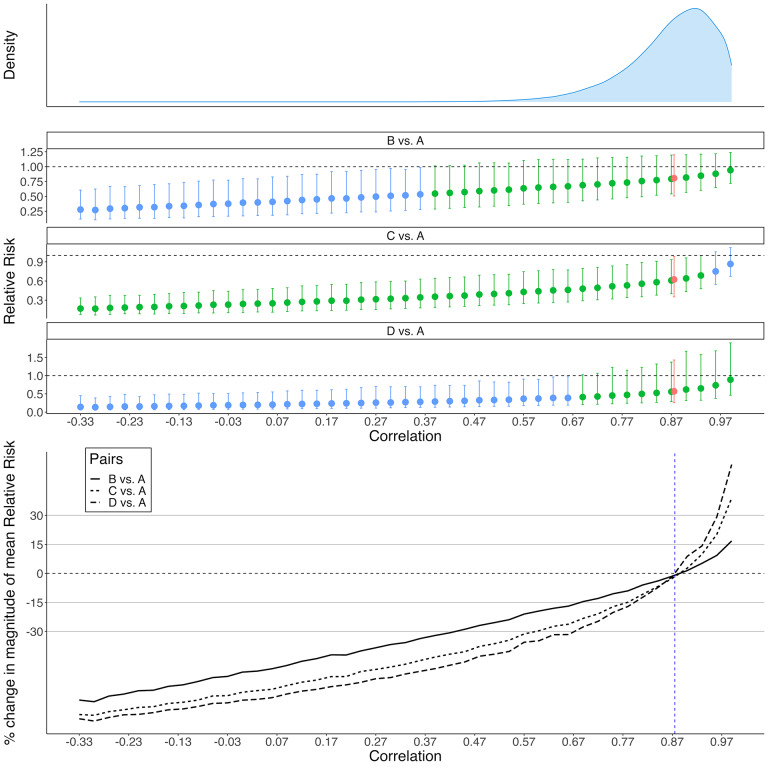
Results of the case study. Note: The plots from top to bottom panels are the density plot of the estimated correlation in Step 1 (Panel I), the plot of the *interval conclusion change* tipping point of the relative risk in three treatment pairs (Panel II), and the plot of *magnitude change* tipping point of the relative risk in three treatment pairs (Panel III). Capital letters indicate the treatment. A = medical therapy; B = PTCA; C = BMS; D = DES. In Panel II, the red color indicates the relative risk estimated in Step 1, the green color indicates that the *interval conclusion* is the same as the conclusion in Step 1, and the blue color indicates that the *interval conclusion* is opposite to the conclusion in Step 1. In Panel III, the vertical line indicates the median correlation at 0.875 estimated in Step 1.

To graphically illustrate the tipping point analysis in the whole range of correlation that makes the covariance matrix positive definite, we examined the case study Trikalinos2009, expanding the *interval conclusion change* and *magnitude change* tipping point search region to encompass the values from 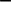 0.33 to 0.99, with adjacent values 0.03 units apart. In the initial analysis, the estimated correlation with a 95% credible interval was 0.875 (0.622, 0.982). Figure 5 displays the plots for both *interval conclusion* change and *magnitude change* tipping points of the RR, along with the density plot of the correlation estimated in Step 1. Note that treatment pairs without an *interval conclusion* change tipping point within the search domain were not shown in the figure.

In the *interval conclusion* change tipping point plot in Figure 5 (Panel II), a transition from green to blue denotes the specific tipping point for each treatment pair. The location of the tipping point also matters: the closer the *interval conclusion change* tipping point relative to the median of the estimated correlation, the more probable that the tipping point may be achieved, and thus the more likely the *interval conclusion change* may occur. Referring to the density plot of the estimated correlation, the order of *interval conclusion* change tipping points for the three treatment pairs, from highest to lowest density, was BMS vs. medical therapy, DES vs. medical therapy, and PTCA vs. medical therapy. This sequence also reflects the probability of an altered *interval conclusion*. Consequently, the RR between BMS and medical therapy was more sensitive to changes in correlation parameters. This made it more likely to shift toward strong evidence supporting the presence of a treatment effect, compared to DES and PTCA. If such a shift occurred, the RR would fall below 1, suggesting a better treatment effect of BMS relative to medical therapy.

In the *magnitude change* tipping point plot, the points where the graphs intersect the horizontal grey lines denote the tipping points for each treatment pair. For instance, considering a threshold at 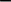 15% and referencing the density plot of the estimated correlation, the *magnitude change* tipping points for the three treatment pairs, in descending order of the density, were DES vs. medical therapy, BMS vs. medical therapy, and PTCA vs. medical therapy. This order implied the potential for a decrease in the population-averaged RR compared to the initial analysis. To fully understand the implications, the *magnitude change* tipping point plot should be interpreted alongside the *interval conclusion* change tipping point plot. For example, while a change in the *interval conclusion* for BMS vs. medical therapy was more probable as the true correlation deviates from the estimated correlation in the AB-NMA model, the magnitude of the mean RR remained fairly stable.

## 5 Discussion

In this article, we proposed a novel tipping point analysis to assess the robustness of treatment effect conclusions, focusing on *interval conclusion* change and *magnitude change*, based on the varying correlation strengths between treatments in an AB-NMA. This proposed tipping point analysis was carried out on 14 NMA datasets, selected based on outcome type, the count of treatments, and the number of studies within each network. Our findings highlighted that occurrences of tipping points, in terms of both *interval conclusion* change and *magnitude change*, may be common. To elaborate more details on the proposed analysis, we focused on one specific NMA dataset as the case study, visually illustrating the tipping point analysis results and interpreting the results in the context of the clinical application. Compared to our previous work on pairwise meta-analysis,^30^ this study extends the concept of tipping point to the more complex framework of NMA. It incorporates more comprehensive considerations of the variance–covariance matrix and introduces a generalized tool for identifying tipping points. The proposed method is also applicable to AB pairwise meta-analysis, as it represents a special case of AB-NMA involving only two treatments within the network.

Our analysis of treatment pairs with an *interval conclusion* change tipping point revealed that the majority (8 out of 13) had limited or no direct comparisons ( 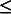 1). This finding highlights the critical role of tipping point analysis in addressing uncertainties in NMAs where direct comparisons among treatments are sparse. By identifying and examining tipping points, researchers can better understand the robustness of the network’s findings and assess how sensitive the results are to assumptions about the correlation. Such insights are particularly valuable in networks with limited data, where reliance on indirect evidence is greater. However, sparsity is not the only factor necessitating this sensitivity analysis. Notably, we observed that three treatment pairs with *interval conclusion* change tipping points had more than 10 direct comparisons. This highlights that tipping point analysis remains useful even in networks where sparsity is not a significant concern. We recommend incorporating the proposed tipping point analysis as a standard component of AB-NMA to complement the findings in treatment effects and comment on the robustness of the conclusion across the possible range of correlation, particularly for networks with sparse direct comparisons.

Moreover, we also recommend incorporating the proposed method for networks with a wide 95% credible interval for the treatment correlation parameter. For such networks, the wide credible intervals indicate substantial uncertainty in the estimation of the correlation structure, and the potential range for encountering a tipping point increases, which reinforces the need for the proposed analysis to supplement an NMA exhibiting that characteristic.

To draw a holistic conclusion, it is also important to consider additional factors. First, clinically meaningfulness should be considered once a tipping point is identified within the high-density region of the correlation parameter. If the assumed correlation value at the tipping point lacks biological plausibility, a robust conclusion may still be reached. Second, while a dichotomized conclusion about the strength of evidence such as the statistical significance and the *interval conclusion* can provide useful insights, it should not be interpreted in isolation but rather considered alongside effect sizes, the length of confidence/credible intervals, and the clinical or practical relevance of the findings. Since the reliance on statistical significance as a sole criterion for drawing conclusions about treatment effects has been widely debated due to its inherent limitations and the arbitrary nature of cut-off values like *p*-value 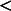 0.05.^38^ We acknowledge this ongoing discussion and emphasize the importance of a holistic approach when interpreting results, particularly in the context of NMA, where multiple assumptions and uncertainties are present. Last but not least, NMA is most justifiable under the evidence consistency assumption, which is defined as the agreement between direct and indirect sources of evidence,^39^ Violating this assumption may result in a biased treatment effect estimation,^40^ and may also impact the conclusion of the proposed sensitivity analysis. Assessment of evidence inconsistency may be considered before conducting the sensitivity analysis.

The proposed approach and our investigation have some limitations that may present opportunities for further expansion in future research. Firstly, our proposed method only focused on the exchangeable correlation structure, assuming a homogeneous correlation across all treatment pairs. This choice was in response to the limitation of sparse data in estimating more complex correlation structures. Consequently, our model may not be applicable to other correlation structures with the heterogeneous correlation assumption.

Secondly, this article focused specifically on the AB-NMA model for binary outcomes. However, the proposed method can be adapted for other types of outcomes with appropriate modifications. For instance, in the context of continuous outcomes, the AB-NMA model framework proposed by Zhang et al.^41^ and Wang et al.^21^ could be adopted and modified, and the null value for the *interval conclusion* would need to be adjusted to align with the evaluation of treatment effects for continuous data.

Thirdly, our method was specifically tailored to a single outcome and focused exclusively on the correlation parameter under a uniform prior. Expanding this approach to accommodate multivariate outcomes in NMAs and incorporating meta-regression frameworks could enhance its applicability and relevance in broader contexts. Additionally, the methodology for identifying tipping points may be transferrable to other key parameters of interest in different settings, providing a valuable sensitivity analysis tool to evaluate the robustness of conclusions across a wide range of applications.

Lastly, the impact of evidence inconsistency on the proposed method is complex and may require further investigation as future work. The impact may depend on the direction of the discrepancy between the direct and indirect evidence, and factors such as the number of studies in each evidence loop within the network, and the presence and degree of heterogeneity,^39^ may also impact the estimation of correlation parameter and need to be carefully accounted for. Moreover, given the inherent challenges of detecting inconsistency due to the low power of available tests and multiplicity of evidence loops,^42^ ^,^ ^43^ empirical datasets alone may be insufficient. Therefore, simulation studies may be necessary to systematically explore the impact of evidence inconsistency under different scenarios.

## Supporting information

Wang et al. supplementary material Wang et al. supplementary material

## Data Availability

The data that support the findings of this study are openly available in the R package “nmadb” at https://cran.r-project.org/web/packages/nmadb/index.html. We also would like to provide a warning on the data discrepancy in the “nmadb” package. We observed certain inconsistencies between the NMA datasets from the “nmadb” package and the data presented in the original papers. For instance, the first author of the Furukawa2014 dataset was mistakenly labeled as Kingdomawa in the “nmadb” package, and there was a discrepancy in the number of studies recorded (48 in “nmadb” vs. 49 in the original paper).

## Supplementary material

The supplementary material for this article can be found at https://doi.org/10.1017/rsm.2025.24.
