# Supplementary material for: Tipping point analysis in network meta-analysis
Source: Res Synth Methods. Author manuscript; Available in PMC 2026 Jun 16. (PMC12527527; doi:10.1017/rsm.2025.24)
Supplement: Supplementary Material [file NIHMS2109496-supplement-Supplementary_Material.pdf]

Appendix 1. JAGS code for Bayesian models

```
###########################################
#### The AB-NMA model (as in tipping point analysis step 1)
   ####
###########################################

model{
 for(i in 1:len){
  logit(p[i]) <- mu[t[i]] + vi[s[i], t[i]]
  r[i] ~ dbin(p[i], totaln[i])
  rhat[i] <- p[i]*totaln[i]
  dev[i] <- 2*(r[i]*(log(r[i]) - log(rhat[i])) +
    (totaln[i] - r[i])*(log(totaln[i] - r[i]) - log(totaln[i] -
        rhat[i])))

 }
 totresdev <- sum(dev[])
 for(j in 1:nstudy){
  vi[j, 1:ntrt] ~ dmnorm(zeros[1:ntrt], T[1:ntrt, 1:ntrt])
 }
 for(j in 1:ntrt){
  AR[j] <- 1/(1 + exp(-mu[j]/sqrt(1 + (16*sqrt(3)
     /(15*3.1415926))^2*pow(sigma[j], 2))))
  mu[j] ~ dnorm(0, 0.001)
  sigma[j] ~ dunif(0.0001, 10)

  # Alternative HHC prior for standard deviation
  #sigma[j] ~ dt(0, a_sd, 1)
 }

 #a_sd ~ dunif(0, 5)

 for(j in 1:ntrt){
  for(k in 1:ntrt){
   T[j,k] <- 1/sigma[j]*1/sigma[k]*ifelse(j == k, diag, offdiag
      )
  }
 }
 diag <- (1 + (ntrt - 2)*rho)/(1 + (ntrt - 2)*rho - (ntrt - 1)*
   rho^2)
 offdiag <- (-rho/(1 + (ntrt - 2)*rho - (ntrt - 1)*rho^2))

 ####################################
```

```
 rho ~ dunif(-1/(ntrt - 1), 0.9999)

 ####################################

 for(j in 1:ntrt){
  for(k in 1:ntrt){
   LRR[j,k] <- log(RR[j,k])
   LOR[j,k] <- log(OR[j,k])
   LOR.med[j,k] <- mu[j] - mu[k]
   RR[j,k] <- AR[j]/AR[k]
   RD[j,k] <- AR[j]-AR[k]
   OR[j,k] <- AR[j]/(1 - AR[j])/AR[k]*(1 - AR[k])
   OR.med[j,k] <- exp(LOR.med[j,k])
   ##############################
   # check Prob(RR>1)
   P1[j,k] <- step(RR[j,k]-1)
   ##############################
  }
 }
 rk[1:ntrt] <- (ntrt + 1 - rank(AR[]))*ifelse(higher.better, 1,
     0) + (rank(AR[]))*ifelse(higher.better, 0, 1)
 rk.med[1:ntrt] <- (ntrt + 1 - rank(mu[]))*ifelse(higher.better
    , 1, 0) + (rank(mu[]))*ifelse(higher.better, 0, 1)
 for(i in 1:ntrt){
  rank.prob[1:ntrt, i] <- equals(rk[], i)
  rank.prob.med[1:ntrt, i] <- equals(rk.med[], i)
 }

}



#########################################################
#### The AB-NMA model with a fixed value of correlation
   parameter rho (as in tipping point analysis step 2) ####
#########################################################

model{
 for(i in 1:len){
  logit(p[i]) <- mu[t[i]] + vi[s[i], t[i]]
  r[i] ~ dbin(p[i], totaln[i])
  rhat[i] <- p[i]*totaln[i]
  dev[i] <- 2*(r[i]*(log(r[i]) - log(rhat[i])) +
    (totaln[i] - r[i])*(log(totaln[i] - r[i]) - log(totaln[i] -
       rhat[i])))
```

```
}
totresdev <- sum(dev[])
for(j in 1:nstudy){
 vi[j, 1:ntrt] ~ dmnorm(zeros[1:ntrt], T[1:ntrt, 1:ntrt])
}
for(j in 1:ntrt){
 AR[j] <- 1/(1 + exp(-mu[j]/sqrt(1 + (16*sqrt(3)
    /(15*3.1415926))^2*pow(sigma[j], 2))))
 mu[j] ~ dnorm(0, 0.001)
 sigma[j] ~ dunif(0.0001, 10)

 # Alternative HHC prior for standard deviation
 #sigma[j] ~ dt(0, a_sd, 1)
}

#a_sd ~ dunif(0, 5)

for(j in 1:ntrt){
 for(k in 1:ntrt){
  T[j,k] <- 1/sigma[j]*1/sigma[k]*ifelse(j == k, diag, offdiag
     )
 }
}
diag <- (1 + (ntrt - 2)*rho)/(1 + (ntrt - 2)*rho - (ntrt - 1)*
   rho^2)
offdiag <- (-rho/(1 + (ntrt - 2)*rho - (ntrt - 1)*rho^2))

####################################

# rho will be assigned a fixed value

####################################

for(j in 1:ntrt){
 for(k in 1:ntrt){
  LRR[j,k] <- log(RR[j,k])
  LOR[j,k] <- log(OR[j,k])
  LOR.med[j,k] <- mu[j] - mu[k]
  RR[j,k] <- AR[j]/AR[k]
  RD[j,k] <- AR[j]-AR[k]
  OR[j,k] <- AR[j]/(1 - AR[j])/AR[k]*(1 - AR[k])
  OR.med[j,k] <- exp(LOR.med[j,k])
  ##############################
  # check Prob(RR>1)
```

```
    P1[j,k] <- step(RR[j,k]-1)
    ################################
   }
  }
  rk[1:ntrt] <- (ntrt + 1 - rank(AR[]))*ifelse(higher.better, 1,
      0) + (rank(AR[]))*ifelse(higher.better, 0, 1)
  rk.med[1:ntrt] <- (ntrt + 1 - rank(mu[]))*ifelse(higher.better
     , 1, 0) + (rank(mu[]))*ifelse(higher.better, 0, 1)
  for(i in 1:ntrt){
   rank.prob[1:ntrt, i] <- equals(rk[], i)
   rank.prob.med[1:ntrt, i] <- equals(rk.med[], i)
  }

}
```

Appendix 2. Supplemental results

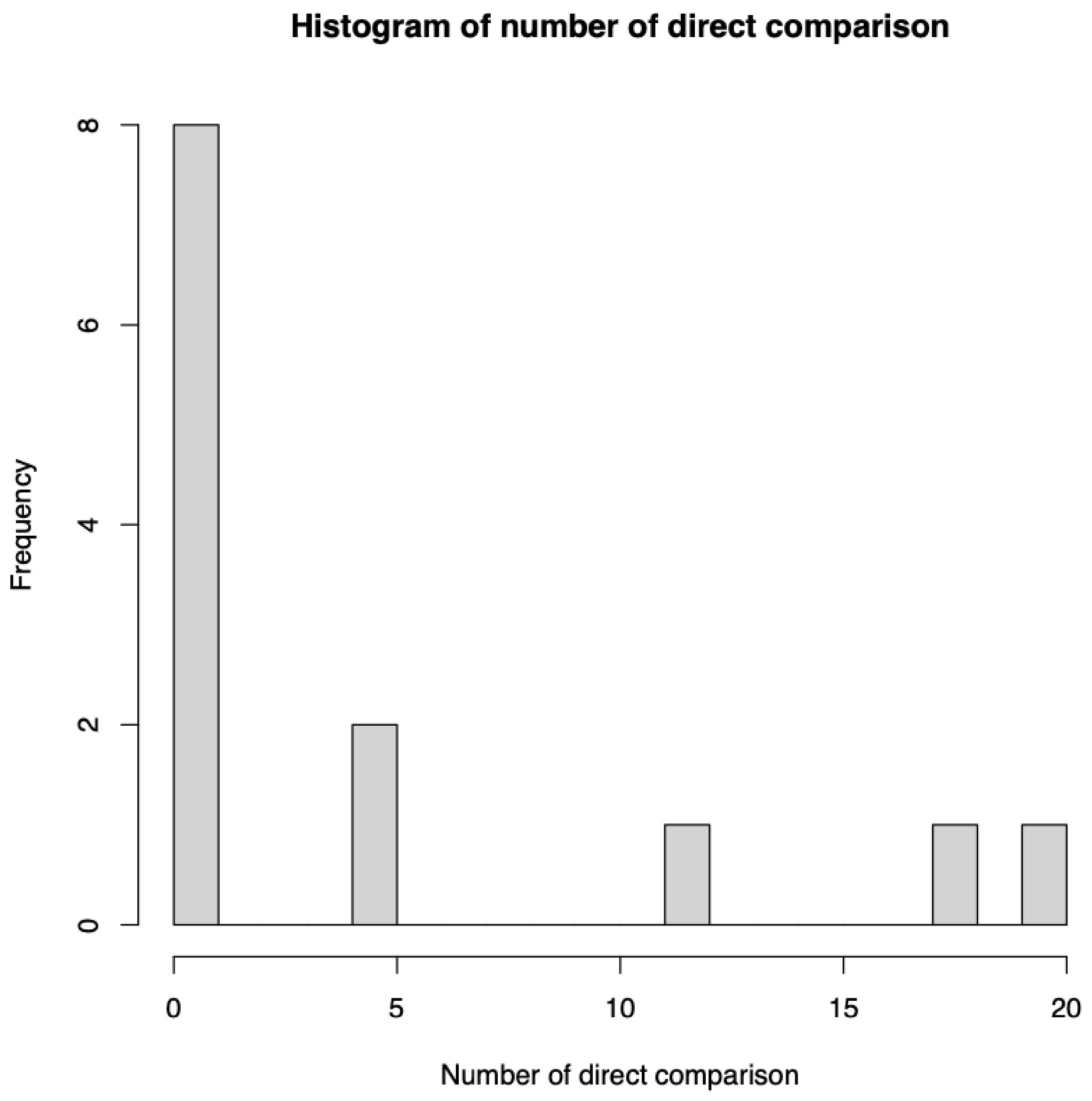

Figure S1: Histogram of the number of direct comparisons among pairs with an *interval conclusion change* tipping point in 14 selected NMA datasets.
